# Stakeholder engagement in health: whose business is it anyway?

**DOI:** 10.11604/pamj.2026.53.134.49911

**Published:** 2026-03-23

**Authors:** Sebastian James Gelderbloem, James Kruger, Brian van Wyk

**Affiliations:** 1 Western Cape Department of Health and Wellness, Cape Town, South Africa,; 2 University of Western Cape, School of Public Health, Cape Town, South Africa

**Keywords:** Stakeholder, engagement, health systems

## Abstract

Universal Health Coverage (UHC) is the cornerstone of equitable health systems and service delivery. Achieving UHC requires both "whole of government" and "whole of society" approaches that are vital for building resilient health systems that promote physical and mental wellness for all communities. In this paper, we review several theories of stakeholder engagement (SE) and argue that meaningful SE - as opposed to stakeholder management - is essential for fostering early participation and democratic decision-making in the transformation of health systems towards UHC. We recommend several key steps to promote early and inclusive SE that actively solicit input from diverse community members, establish open platforms for dialogue, and leverage the expertise and resources of various sectors. By creating a collaborative environment that includes each individual, whether an educator, policymaker, family member, or business leader, amongst others, we can advance the goals of UHC and build health systems that benefit everyone.

## Essay

Universal Health Coverage (UHC) is key to ensuring quality, affordable healthcare for all [1]. The WHO defines UHC as the principle that all individuals and communities should receive the health services they need without facing financial hardship. According to the WHO, achieving UHC necessitates the participation of multiple stakeholders, including national and local governments, international organizations, and civil society. Furthermore, effective implementation of UHC requires a multidisciplinary approach that integrates health systems research, policy reforms, and multi-stakeholder engagement to address the diverse needs of populations. The "business" of health aimed at achieving UHC involves a complex interplay between public and private sectors, with governments playing a crucial role in ensuring equitable access to health services. The notion of “health is everybody´s business” [2] implicates a shared responsibility to build health systems' resilience and support for the health and mental wellbeing of the population. This shared responsibility can manifest through the integration of the 'whole of government´ and the 'whole of society´ approaches, where government and civil society work together in creating public value as a unified goal, i.e., an “equity agenda”. An equity agenda necessitates public policy reform to support collective action, collaboration, innovation, societal dynamics, and ultimately the well-being of communities. In answering the call to “health is everybody´s business”, it is imperative to review our understanding of stakeholder engagement (SE) and to consider how to meaningfully engage with stakeholders to further an equity agenda for health.

Early notions of engaging with stakeholders appeared in the 1990s, but “stakeholder engagement” as a construct only gained prominence in the stakeholder literature at the beginning of the 2000s. The construct was introduced to make a distinction between the “one-sided management of stakeholders” and to delineate between “enhancing shareholder value” and “engaging stakeholders for long-term value creation” [3]. Stakeholder theory places stakeholders at the center of strategic thinking and positions relationships with stakeholders as a focus of analysis. Although it is widely accepted that SE is distinct from stakeholder management, some diffusion persists in the literature, and sometimes SE is used as a variation of stakeholder management. In contrast to the idea of reciprocity innate in SE, stakeholder management is inherently unilateral because when organizations conventionally manage their stakeholders, they “take steps to defend themselves from the demands of stakeholders” [4]. In contrast, genuine SE involves interactions between identified groups of people and provides stakeholders with an opportunity to discuss needs, raise their concerns and opinions, and ensures that this information is taken into consideration when making health policy, strategic, and operational decisions.

**Current models of stakeholder engagement:** various SE models exist with the aim to foster inclusivity, collaboration, and alignment among diverse groups to address local challenges in sectors like healthcare, education, and infrastructure. The multi-stakeholder model involves governments, businesses, civil society, and local communities to maximize the impact of pooled resources and expertise, as seen in the United Nations' Sustainable Development Goals framework [5].

The public-private partnerships (PPP) model leverages the strengths of both government and private sector resources to enhance infrastructure and services in areas where government capacity may be limited. This approach has proven effective in energy and healthcare projects, such as the Coal India and *Électricité de France* (EDF) joint venture in 2025 [6]. By combining resources and expertise, PPPs can deliver essential services more efficiently and sustainably.

In contrast, the community-centered model emphasizes local participation to ensure that proposed solutions are sustainable and culturally relevant. Engaging community members in the decision-making process fosters ownership and accountability [7]. By prioritizing local input, this model enhances initiative effectiveness and ensures that interventions resonate with the specific needs and values of the community. The inclusive development model focuses on integrating marginalized groups into decision-making processes to reduce inequalities. By actively involving underrepresented populations, this model addresses systemic barriers and promotes social equity, ensuring that diverse voices influence policies and programs that impact their lives [8]. This approach not only fosters inclusivity but also enhances the overall effectiveness and sustainability of development initiatives.

Similarly, the quadruple helix model connects government, industry, academia, and civil society to promote innovation and transparency, particularly in sectors like healthcare and agriculture in rapidly developing countries such as Kenya and India [9]. By leveraging diverse expertise and resources, this model effectively addresses local challenges and ensures a variety of perspectives are included in decision-making processes.

The participatory governance model, on the other hand, empowers citizens to take an active role in decision-making, as seen in Brazil, where community members directly influence the allocation of a portion of the municipal budget. This approach enhances transparency and accountability while challenging conventional top-down governance structures, making it exceptional in amplifying marginalized voices [10]. Both the quadruple helix and participatory governance models are considered "exceptionalist" due to their adaptability and capacity to address contextual needs and power dynamics often overlooked by traditional frameworks. The flexibility of the quadruple helix model allows for tailored solutions to local challenges, while the participatory governance model disrupts established hierarchies, fostering social equity. Additionally, the corporate social responsibility (CSR) model encourages corporations to contribute to local development through investments in social services. This model underscores the role of businesses in promoting community well-being by aligning operations with societal needs. By promoting collaboration across sectors and elevating community voices, these frameworks represent significant departures from conventional SE strategies, offering innovative approaches to complex societal issues.

**Challenges with stakeholder engagement:** stakeholder engagement (SE) is often experienced as complex and muddy due to the diverse nature of stakeholders, which include government agencies, non-governmental organizations, communities, healthcare providers, and private sector entities. Each stakeholder group may have different goals, resources, and levels of influence, leading to intricate dynamics and potential conflicts of interest. Additionally, stakeholders may possess varying levels of power and authority, which further complicates the engagement process. Health is everybody´s business, and collaborative engagement across government departments and society presents challenges due to the notions of where partners perceive the responsibility to lie, as well as negotiating competing priorities. There is a default position (assumption) that the health sector needs to tend to health (care) issues and, therefore, maintain a siloed approach in governance, strategy, operationalization, and implementation of health services. Health departments typically provide a technical response to health issues, but it has become evident through the COVID-19, TB, and HIV/AIDS pandemics that health responses must be accompanied by equally equitable social responses. Technical health responses have typically led to one-sided development of policies and programmes that are experienced as exclusionary by partners and communities, and therefore not aligned to facilitate meaningful community participation. There are many examples where poorly implemented SE resulted in a lack of understanding in communities, preferences, and the ultimate failure of health campaigns.

The barriers to effective SE are well noted and include the following: 1) a lack of coordination: with numerous stakeholders involved, coordinating efforts and ensuring alignment towards common goals can be challenging. Without effective coordination mechanisms, stakeholders may work in silos, leading to duplication of efforts and inefficiencies; 2) communication challenges: effective communication is crucial for SE, yet it can be hindered by differences in language, culture, and communication styles among stakeholders. Miscommunication or misunderstandings can impede collaboration and trust-building efforts; 3) power imbalances: power differentials among stakeholders can influence decision-making processes and outcomes. Certain stakeholders, such as government agencies or large corporations, may wield more power and influence than others, potentially marginalizing less powerful stakeholders and limiting their participation in decision-making processes; 4) resource constraints: limited resources, both financial and human, can hinder stakeholders' ability to actively participate in engagement activities. This can particularly affect marginalized or under-resourced communities, exacerbating inequities in the engagement process; 5) resistance to change: stakeholders may be resistant to engaging in collaborative processes or implementing changes that could potentially disrupt the status quo or threaten their interests. Overcoming resistance to change requires effective communication, trust-building, and stakeholder buy-in.

Addressing these barriers requires a comprehensive approach that prioritizes inclusive participation, transparent communication, and equitable decision-making processes. Establishing clear frameworks for SE, fostering a culture of collaboration and trust, and providing adequate resources and support for stakeholders can help mitigate the complexities and challenges associated with SE in the health sector.

**Figure 1:**
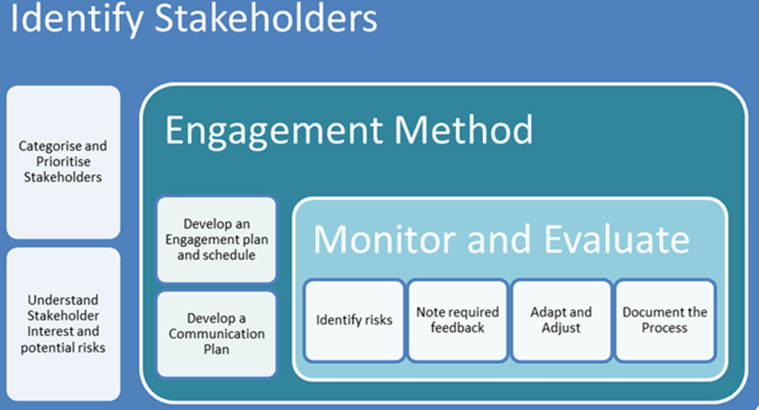
steps in stakeholder engagement

**A new approach to improving stakeholder management:** Figure 1 outlines a proposed approach to SE and management that would foster trust, promote inclusivity, and potentially lead to better sustained outcomes (Figure 1).

**Step 1: identify and prioritize stakeholders:** engaging the right stakeholders from the beginning ensures that efforts are targeted, efficient, and aligned with the needs of those who have the most influence on or are most affected by healthcare programs and who can add value. Identifying key stakeholders, understanding their interests, and recognizing their motivations and challenges helps create a more inclusive decision-making process.

Actionable steps: map out all potential stakeholders considering the definition of a stakeholder (community leaders, patients, healthcare providers, policymakers, private sector partners, etc.); categorize and prioritize them based on their level of influence, interest, and impact on healthcare programs or projects; compile an assessment on each of the prioritized stakeholders identifying their interest and the potential barriers and/or risk they may pose before engaging in discussions.

**Step 2: develop an engagement strategy and outline clear engagement processes:** develop an engagement plan: an engagement plan provides clear direction and sets a framework within which to operate; develop a communication strategy: a communication plan is essentially to ensure clear, consistent, and effective interactions between all relevant parties. A well-structured communication plan helps align messaging, manage expectations, and foster transparency, ultimately building trust and collaboration among stakeholders and minimizing misunderstandings. Build trust and foster long-term relationships: trust is the foundation of any successful engagement, and without trust, stakeholders may resist participation, leading to low program/project adoption or collaboration and ineffective healthcare services. Trust can be established through transparency, culturally appropriate communication, and consistent interaction over time.

Actionable steps: engage community leaders as intermediaries to facilitate discussions; use culturally relevant messaging, such as local languages and traditions, to improve comprehension and relatability; ensure transparency in decision-making by openly sharing information and updates; establish a long-term commitment to stakeholder engagement rather than treating it as a one-time consultation; establish clear mechanisms for recognition and engagement.

Co-design and set collective goals: a collaborative goal-setting approach ensures that healthcare programs/projects align with the actual needs of the community and other stakeholders. According to stakeholder democracy principles, involving stakeholders in governance and decision-making improves accountability and program/project effectiveness.

Actionable steps: organize participatory workshops where stakeholders collectively define program objectives and outcomes; encourage shared decision-making to foster a sense of ownership and commitment; address concerns and objections early by providing evidence-based options and alternative solutions; align collective goals with measurable outcomes to track progress effectively.

Manage power dynamics and provide incentives for participation: power imbalances can hinder effective SE, as some groups (e.g., policymakers, private-sector players) may dominate discussions while marginalized voices (e.g., underserved communities) go unheard. Managing these dynamics is essential to ensure fair participation and inclusivity.

Actionable steps: create structured discussion frameworks that give equal opportunity for all stakeholders to contribute; partner with local organizations to empower underrepresented groups; offer incentives such as free health screenings, educational materials, or digital tools that encourage participation; utilize digital platforms and social media to amplify community voices and collect broad-based feedback.

**Step 3: monitor and evaluate engagement:** sustainable engagement requires ongoing monitoring and evaluation of communication, risks, interaction, and progress. Regular feedback loops ensure that stakeholders remain informed, involved, and motivated to participate. Failing to document engagements, adapt when required, and respond to challenges can lead to programme or project failure, particularly SE.

Actionable steps: establish multiple channels for feedback, including surveys, focus groups, and digital tools; schedule regular updates and reports on program progress and stakeholder input; adapt engagement strategies based on stakeholder feedback to continuously improve initiatives; hold quarterly or biannual review meetings to evaluate program impact and adjust strategies accordingly.

**Conclusion:** stakeholders are critical partners to national departments of health and have huge impacts on health service delivery and outcomes. However, due to multiple factors such as resource constraints, lack of awareness, bureaucratic inertia, fear of conflict, insufficient skills and training in SE, perceived irrelevance, SE complexity, and short-term focus, SE and maintenance of long-term relationships are most often not prioritized. Addressing these factors requires a shift in perspective, emphasizing the long-term gains of comprehensive engagement in building public trust, achieving sustainable outcomes, and enhancing policy effectiveness, hence realizing the “health is everybody´s business” notion. To overcome these challenges, it is imperative to adopt a paradigm shift that recognizes the strategic importance of sustained SE.

The new paradigm of SE emphasizes thoughtful planning, agreement, active listening, and responsiveness, with a strong focus on building trust. This approach is particularly transformative in the realm of health service delivery. By fostering robust relationships with stakeholders, including patients, healthcare providers, and community organizations, health initiatives can be more effectively aligned with community needs and expectations. Ultimately, the success of health initiatives hinges on this collaborative effort, which not only enhances service delivery but also empowers communities to take ownership of their health outcomes. In a rapidly evolving health landscape, effective SE is not just beneficial; it is essential for achieving UHC and ensuring sustainable health systems that truly serve the people.
